# STING-Triggered CNS Inflammation in Human Neurodegenerative Diseases

**DOI:** 10.3390/biomedicines11051375

**Published:** 2023-05-05

**Authors:** Alex S. Ferecskó, Miranda J. Smallwood, Adrian Moore, Corin Liddle, Jia Newcombe, Janet Holley, Jacqueline Whatmore, Nicholas J. Gutowski, Paul Eggleton

**Affiliations:** 1 UCB Pharma, Slough SL1 3WE, UK; 2 Institute of Biomedical and Clinical Science, University of Exeter Medical School, Exeter EX1 2LU, UK; 3 Bioimaging Unit, University of Exeter, Geoffrey Pope Building, Exeter EX4 4QD, UK; 4 NeuroResource, UCL Queen Square Institute of Neurology, London WC1N 1PJ, UK; 5 Revolo Biotherapeutics, New Orleans, LA 70130, USA

**Keywords:** cerebral endothelial cells, cGAS, cortical neurons, neuroinflammation, palmitic acid

## Abstract

Background: Some neurodegenerative diseases have an element of neuroinflammation that is triggered by viral nucleic acids, resulting in the generation of type I interferons. In the cGAS-STING pathway, microbial and host-derived DNA bind and activate the DNA sensor cGAS, and the resulting cyclic dinucleotide, 2′3-cGAMP, binds to a critical adaptor protein, stimulator of interferon genes (STING), which leads to activation of downstream pathway components. However, there is limited work demonstrating the activation of the cGAS-STING pathway in human neurodegenerative diseases. Methods: Post-mortem CNS tissue from donors with multiple sclerosis (*n* = 4), Alzheimer’s disease (*n* = 6), Parkinson’s disease (*n* = 3), amyotrophic lateral sclerosis (*n* = 3) and non-neurodegenerative controls (*n* = 11) were screened by immunohistochemistry for STING and relevant protein aggregates (e.g., amyloid-β, α-synuclein, TDP-43). Human brain endothelial cells were cultured and stimulated with the STING agonist palmitic acid (1–400 μM) and assessed for mitochondrial stress (release of mitochondrial DNA into cytosol, increased oxygen consumption), downstream regulator factors, TBK-1/pIRF3 and inflammatory biomarker interferon-β release and changes in ICAM-1 integrin expression. Results: In neurodegenerative brain diseases, elevated STING protein was observed mainly in brain endothelial cells and neurons, compared to non-neurodegenerative control tissues where STING protein staining was weaker. Interestingly, a higher STING presence was associated with toxic protein aggregates (e.g., in neurons). Similarly high STING protein levels were observed within acute demyelinating lesions in multiple sclerosis subjects. To understand non-microbial/metabolic stress activation of the cGAS-STING pathway, brain endothelial cells were treated with palmitic acid. This evoked mitochondrial respiratory stress up to a ~2.5-fold increase in cellular oxygen consumption. Palmitic acid induced a statistically significant increase in cytosolic DNA leakage from endothelial cell mitochondria (Mander’s coefficient; *p* < 0.05) and a significant increase in TBK-1, phosphorylated transcription factor IFN regulatory factor 3, cGAS and cell surface ICAM. In addition, a dose response in the secretion of interferon-β was observed, but it failed to reach statistical significance. Conclusions: The histological evidence shows that the common cGAS-STING pathway appears to be activated in endothelial and neural cells in all four neurodegenerative diseases examined. Together with the in vitro data, this suggests that the STING pathway might be activated via perturbation of mitochondrial stress and DNA leakage, resulting in downstream neuroinflammation; hence, this pathway may be a target for future STING therapeutics.

## 1. Introduction

Most neurodegenerative diseases (NDs) have an element of neuroinflammation that has been speculated to be triggered by viral nucleic acids, resulting in the generation of type I interferons. The cytosolic DNA sensor cyclic GMP–AMP synthase (cGAS)-stimulator of interferon genes (STING) pathway was initially thought to be activated primarily by viral DNA [1]. However, more recent evidence suggests that self-DNA can activate the pathway [2], and subsequent production of the cyclic dinucleotide, 2′3-cGAMP, binds to the critical adaptor protein STING, resulting to the activation and translocation to the trans-Golgi network. This leads to recruitment of the interferon inducer—TANK-binding kinase 1 (TBK-1)—and phosphorylation of IRF3 and IKK, resulting in the production of type I interferons (IFNs) and pro-inflammatory cytokines. There is limited work demonstrating the presence of cGAS-STING activation in primary cells of neurodegenerative CNS tissue. Despite this, the sensing of endogenous DNA and type I IFNs is emerging as important for the development of neurodegenerative diseases. The STING and TBK-1 are implicated in several neurodegenerative conditions, and patients have shown increased expression of cGAS-STING pathway genes in their neuronal tissues [3,4,5].

Thus, investigations of cGAS-STING IFN-driven diseases, expanding beyond the more systemic inflammatory and infectious diseases, are more commonly encountered [6,7]. Indeed, type I IFN production is believed to influence neurodegenerative progression [8]. When the blood–brain barrier (BBB) becomes compromised during neuroinflammation, type I IFN can directly enter the CNS [9]. Neurotrophic viruses in the systemic circulation often encounter the first assault of IFN from dendritic cells. If this fails, there is a need for further production of IFN. The requirement for locally produced type I IFNs at the gateway to the BBB and CNS may arise from the necessity to have rapid innate immunity for suppressing viral infections that avoid systemic elimination. There is evidence that human cerebral microvascular endothelial cells (hCMEC/D3) [10] and CNS cells such as neurons, astrocytes and microglia also produce IFNs [11].

Type I and II interferons are cytokines produced as part of our anti-viral immune response. The type I interferons comprise of a group of 13–14 cytokines, together with IFN-β, that can signal via the same type I interferon receptors. Type II interferon is known as IFN-γ and is generated predominantly by natural killer cells and signals via an IFN-γ receptor. They all have important separate and overlapping roles against viruses and other microbes [12]. In general, cGAS stimulation preferentially elicits an IFN-β response, whereas Toll-like receptor stimulation predominately increases IFN-α expression [13]. Recent studies have implicated type I IFN-dependent signalling as an inflammatory driver in certain NDs [14,15]. Cellular stress generated by a build-up of toxic protein aggregates in neural and brain endothelial cells can lead to the release of mitochondrial and nuclear dsDNA into the cell cytosol, where it may act as a self-ligand, activating the STING agonist γ-interferon-inducible protein (IFI16) and cGAS [16,17]. Indeed, the expression of STING and cGAS has been demonstrated in human astrocytes and microglial cells [18].

Although other studies have focused on the activation of cGAS-STING in glial cells, there is less information about whether the brain endothelial cells or neurons participate in STING activation. Here, we show that in human CNS tissue, robust levels of STING are present and higher in the brain microvascular and neuronal cells of several NDs, including Alzheimer’s disease (AD), amyotrophic lateral sclerosis (ALS), Parkinson’s disease (PD) and multiple sclerosis (MS), compared to non-neurodegenerative samples. Palmitic acid (PA) is a common saturated fatty acid that serves as an energy source, but it can also cause lipotoxicity during higher intake over long periods [19]. Additionally, PA can cause a pro-inflammatory microenvironment, leading to BBB leakage, subsequently entering the brain and causing harmful effects to neurons and glial cells [20,21,22,23]. The precise mechanism of PA-induced damage to the brain is not known. In vitro studies suggest that PA can induce α-synuclein accumulation [24], tau hyperphosphorylation and β-amyloid protein (Aβ) formation [25].

In our study, we investigated the abundance of cGAS-STING pathway components in neural cells with a build-up of protein aggregates in brain tissue from four human neurodegenerative diseases. Moreover, we investigated the effect of cell stress induced by PA on mitochondrial DNA release from brain endothelial cells that can evoke a change in protein homeostasis, that triggers activation of components of the cGAS-STING innate immune response.

## 2. Materials and Methods

### 2.1. Study Approval and Human Tissue

Our study used human brain or spinal cord post-mortem tissues from 16 patients with neurological diseases (multiple sclerosis (*n* = 4), Alzheimer’s disease (*n* = 6), Parkinson’s disease (*n* = 3) and spinal cord from amyotrophic lateral sclerosis (*n* = 3)) and 11 control patients with a non-neurological background. Both diseases and control tissues were obtained, with ethical approval, from tissue banks. A total of 94 sections were immuno-stained and used for qualitative observations (Supplementary Table S1). All tissues were obtained with Material transfer agreements from the UK MS Society Tissue Bank, NeuroResource tissue bank and Queen Square Brain Bank for Neurological Disorders London, UK (see Institutional Review Board Statement below for more details). 

### 2.2. Antibodies and Reagents

For immunoblotting of cGAS-STING and downstream pathway proteins, the primary antibodies used were mouse monoclonal anti-human STING (R&D Systems, Abingdon, UK—MAB7169), rabbit polyclonal anti-human cGAS (Abcam, Cambridge, UK—ab224144), mouse monoclonal anti-human TBK1 (Cell Signalling Technology, Danvers, MA, USA—#3504) and mouse monoclonal anti-human ICAM-1 (Abcam, Cambridge, UK —ab2213). The appropriate infrared dye secondary antibodies used were goat anti-rabbit green (Li-COR Biosciences, Cambridge, UK—926-32211 800CW) and goat anti-mouse- green (Li-COR Biosciences, Cambridge, UK—926-32210 800CW). For immunofluorescence analysis of STING distribution in disease and control CNS tissues, sections were probed with a number of primary antibodies, including mouse monoclonal anti-human STING (R&D Systems, Abingdon, UK—MAB7169), rabbit anti-human amyloid-β (Abcam, Cambridge, UK—ab2539), rabbit monoclonal anti-human TDP-43 (Abcam, Cambridge, UK—ab109535), rabbit monoclonal anti-human α-synuclein (Abcam, Cambridge, UK—ab209538) and mouse monoclonal anti-human calnexin (ThermoFisher, Paisley, UK—MA3-027). For cell-type-specific identification and co-localisation of STING with distinct neural cell types and brain endothelial cells, the following primary antibodies were used: rabbit anti-human NeuN (Abcam, Cambridge, UK—ab177487), rabbit anti-human GFAP (Thermo Fisher PA5-16291) and rabbit anti-human CD68 (ThermoFisher, Paisley, UK—PA5-32330), and the corresponding secondary antibodies goat anti-rabbit Alexa fluor 488 (ThermoFisher, Paisley, UK—A-11024) and goat anti-mouse Alexa fluor 647 (ThermoFisher, Paisley, UK—A-21235) (Supplementary Table S2).

PA was purchased commercially for the preparation and treatment of STING pathway agonist experiments on cultured cells (Sigma, Dorset, UK—PO500). Saturated PA, a lipid-containing medium, was prepared by combining PA with bovine serum albumin (BSA). Briefly, PA was dissolved in ethanol to produce a 200 mM solution, and then combined with 20% free fatty acid-free, low-endotoxin BSA (Sigma, Dorset, UK—1595) to produce stock solutions with PA concentrations ranging from 1 to 4 mM. These stock solutions were filter-sterilised and stored at −20 °C. A control medium containing ethanol and BSA was also prepared. Fresh working solutions were prepared by diluting each stock solution (1:10) in the culture medium. The final BSA concentration was kept constant in all PA working solutions, whereas the PA-to-BSA ratio varied with the PA concentrations. All cells tested were cultured and grown to confluency at 4% oxygen in an oxygen-regulated hypoxia chamber (Baker Ruskinn, Bridgend, UK). Cells were then exposed to PA (between 1 and 400 μM) or control media for 16 h.

### 2.3. Histology and Immunohistochemistry of Human CNS Tissues

For immunofluorescent staining, snap-frozen post-mortem brain and spinal cord tissue sections were screened by immunohistochemistry using specific antibody probes for STING, appropriate pathological aggregates of protein (e.g., amyloid-β, α-synuclein or TDP-43) and marker-specific antibodies directed against neural cell types (e.g., microglia: anti-CD68, astrocyte: anti-GFAP and neurons: anti-NeuN). Glass-mounted tissue sections (10 μm) were rapidly fixed in 4% PFA and incubated at room temperature with primary and relevant secondary antibodies in PBS at a 1:100 concentration for 4 and 1 h, respectively (Supplementary Table S2). No additional antigen retrieval steps were used. Autofluorescence eliminator (Millipore, Temecula, CA, USA—2160) was added to the tissue for 5 min and washed off in successive 70% ethanol washes. Tissue sections were washed, rehydrated and mounted in anti-quenching fluorescent mounting medium with 4′,6-diamidino-2-phenylindole (DAPI—ProLong™ Gold anti-fade mountant, Fisher Scientific, Paisley, UK—P36931). Coverslips were sealed with nail varnish and stored at 4 °C in the dark for subsequent microscopic examinations. Regions of interest of characteristic neuronal structures in brain and spinal cord tissue were identified by Nissl stain on tissue sections adjacent to the same tissue block that was used for subsequent triple immunofluorescent labelling. For the standard Nissl stain, Cresyl violet acetate solution (Sigma, Dorset, UK—C5042) was used with steps of alcohol differentiation (70% with acetic acid), and the sections were assessed based on nuclear structures and weakly labelled perikarya.

### 2.4. Microscopy

Immunofluorescent detection of labelled CNS sections was carried out using a Leica DM4000 B LED fluorescence microscope equipped with a black and white digital camera (Leica Microsystems, Milton Keynes, UK). High-resolution pseudo-colour images were captured using air (20×, 40×) and oil immersion objectives (60×). To assess co-localisation between distinct immunosignals, collages of images were captured of each of the three channels over selected areas in cortical grey matter (AD), white matter (MS), SnPC (PD) or the cervical spinal cord (ALS) of both the diseased tissue sections and the corresponding control tissue samples. A built-in software algorithm was used to visualise co-localisation at the cellular level with the aid of specific marker antibodies to distinguish between neural cell types.

To minimise the fading of immunofluorescence and, therefore, altering the intensity of the immune signal, care was taken to complete micro-photographing in one session and to avoid exposing the sections for prolonged periods to UV light or to repeat the capturing process on the same areas of the sections. To estimate the intensity of immunolabelling and provide a comparable dataset for qualitative assessment, exposure parameters (illumination strength, exposure time, etc.) were kept constant and preserved throughout the entire image-taking process. Image taking was performed only once on a specified area to prevent false positive and negative results due to photobleaching and/or overexposure.

Cultured and stained hCMEC/D3 cells were observed and imaged using a Zeiss (Oberkochen, Germany) Elyra 7 lattice-structured illumination microscope (SIM; Zeiss Zen Black 3.0), equipped with a Plan-Apochromat 60×/1.4 Oil DIC M27 objective. The dual-PCO edge sCMOS cameras were set to collect 1024 × 1024 pixels for a frame size of 64 × 64 μm, which resulted in a pixel scaling of 0.031 μm. Z-stacks with a range of 6–10 μm were selected, with an optical section of 90–100 nm, which meets Nyquist sampling requirements.

The Elyra 7 was configured to use a quad-core main beam splitter (LBF 405/488/561/642), and two different camera filter sets were used for 405 and 488 and 561 wavelengths, respectively (BP 420–480 + BP 495–550 and BP 570–620 + LP 655). Several prerequisites for accurate co-localisation measurements were checked before imaging all treatments. In brief, the dynamic range was setup to be suitable for all treatments (>1000). The image quality was assessed for high noise levels and bleed-through, and the system point spread function (PSFs) was checked to make sure it met the specifications. The processed images were examined for any significant artefacts.

The sample was excited with a 405 and 488 nm (100 mW) laser and a 561 nm (100 mW) laser, with the power set to 20% for all tracks. The SIM imaging parameters were as follows: the number of SIM phases = 13, and the SIM grating periods varied according to the excitation wavelength, from 34.0 to 42.0 μm.

All images were then SIM-processed using standard strength computation settings for fixed samples within Zen black 3.1 (Zeiss, Cambourne, UK). Processing was performed on an Aquifer HIVE-multicore processing workstation. Background levels of fluorescence were obtained by measuring the mean intensity of each stain outside the cells. Images were then linearly spectrally unmixed (LSU) to remove background and eliminate any crosstalk that the system’s main beam splitters and filters had not addressed. LSU was performed using automatic two-component extraction (Zeiss Zen Blue 2.3).

### 2.5. Human Brain Microvascular Endothelial Cell (hCMEC/D3) Culture

The blood–brain barrier endothelial cell line, human cerebral microvascular endothelial cells (VHBio Ltd., Gateshead, UK—hCMEC/D3 cells), was cultured and grown to confluence in rat-tail collagen type I-coated (100 μg/mL) tissue culture flasks at 37 °C and in a 4% O_2_/5% CO_2_/nitrogen humid atmosphere. Cells were cultured in PromoCell MV-2 endothelial cell growth medium, MV-2 supplementMix (Merck, Dorset, UK—C-39226), with 10% heat-inactivated foetal bovine serum and gentamicin (50 μg/mL). The hCMEC/D3s were used for up to 40 passages. Confluent cells were trypsinised using 0.05% *v*/*v* trypsin/EDTA, and then passaged and sub-cultured.

### 2.6. Immunoblot Analysis for cGAS, STING, TBK-1, Phospho-IRF3 and ICAM-1

Whole-cell protein isolates were collected from hCMEC/D3s using RIPA lysis buffer (Fisher Scientific, Loughborough, UK—89901) at 4 °C with protease inhibitor tablets (Fisher Scientific, Loughborough, UK—10668304,), phosphatase inhibitor cocktail (Fisher Scientific, Loughborough, UK—15614189) and analysed by immunoblotting. Samples were electrophoresed on 12% mini-Protean TGX SDS-polyacrylamide gradient gels (Bio-Rad, Watford, UK ) in running buffer (25 mM Tris, 192 mM Glycine, 0.1% SDS, pH 8.3) at a constant voltage of 75 V for 15 min, and then 100 V for 35–45 min. Separated proteins were transferred to premade trans-blot 0.2 μm nitrocellulose membranes (Bio-Rad) using a Trans-Blot Turbo™ system (BioRad, Watford, UK—1704150) at 1.3 mA/25 V for 3 min. Blotted membranes were washed in water, and total protein loading was assessed using Revert™ 700 Total Protein Stain (Li-COR Biosciences, Cambridge, UK—926-11010) and imaged using an Azure Biosystems C500 Infrared imaging system. Blots were incubated in and blocked from non-specific antibody binding with TBST with 3% *w*/*v* BSA and 0.1% *v*/*v* Tween for one hour, and then incubated overnight at 4 °C with a rabbit polyclonal antibody directed against human cGAS (Abcam, Cambridge, UK—ab224144; 1:1000 dilution), a mouse monoclonal antibody against human STING (R&D Systems, Abingdon, UK—MAB7169; 1:1000 dilution), mouse monoclonal anti-human ICAM-1 (Abcam, Cambridge, UK—ab53013 MEM-11), a rabbit monoclonal antibody directed against human TBK1 (Cell Signalling Technology, Danvers, MA, USA—3504; 1:1000 dilution) or phosphoIRF3 ser396 (Cell Signalling Technology, Danvers, MA, USA—4947; 1:1000 dilution—rabbit mAb 4947). After 3 washes in TBS-Tween (Tris, 20 mM, NaCl, 150 mM, pH 7.6, 0.1% *v*/*v* Tween), blots were probed with either of the two Li-COR Biosciences secondary infrared antibodies described above (green anti-mouse or anti-rabbit) at 1:15,000, diluted in blocking buffer at RT for 1 h. Blots were digitally imaged using a Li-COR Biosciences scanner (Odyssey CLx Imager). Image studio software (Li-COR Biosciences) was used to normalise the target band intensity from total protein loading images and using lane normalisation factors. In addition, a rabbit anti-human β-actin antibody (Cambridge Bioscience—rabbit A300-485A, 1:3000 dilution, or Merck, Dorset, UK—mouse anti-human A5316, 1:3000 dilution) was used to confirm equal loading. The immunoblots shown are representative of at least three separate experiments.

For the post-transfer protein normalisation measurement, 5 mL of Revert™ 700 protein stain (Li-COR Biosciences) was added to each blot for 5 min RT, then briefly washed with 6.7% *v*/*v* acetic acid and 30% *v*/*v* methanol in H_2_O. Stained membranes were imaged in the 700 nm channel with an Azure Biosystems C500 Infrared imaging system. The lane with the most protein was assigned a value of 1 and all others a fraction of that to obtain the lane normalisation factor (LNF): the signal for each lane/signal for the lane with the highest signal. Normalised signal = target band signal/LNF.

### 2.7. IFN-β Detection in hCMEC/D3 Supernatants by ELISA

A commercial ELISA assay was purchased from R&D systems (Human IFN-β ELISA, Kit 41410) and employed to measure IFN-β in endothelial cell culture supernatants pre- and post-stimulation with 1–400 μM of PA, following the manufacturer’s instructions.

### 2.8. Detection of Cytosolic DNA and Mitochondria Staining Procedures

Cytochemical staining was performed with hCMEC/D3 cells grown in eight-well chamber slides (Lab-Tek 154534). Cytosolic DNA and mitochondria staining in live cells were achieved using the protocol of Ashley and colleagues [26]. A diluted stock of PicoGreen solution (Molecular Probes, Eugene, OR, USA) at 3 μL/mL was added directly into the cell culture medium. The hCMEC/D3 cells were then incubated for 1 h at 37 °C, unless stated otherwise, under standard culture conditions. In some experiments, we co-stained hCMEC/D3 cells with the mitochondria selective dye Mitotracker Red CM-H2XRos (Molecular Probes, Eugene, OR, USA) by adding 100 nM directly to the culture medium and incubating the cells for 2–15 min, followed by incubation in dye-free medium (phenol red-free DMEM supplemented with 4.5 g/L of glucose and 25 mM of HEPES buffer) for a further 20 min. The cells were rinsed three times in pre-warmed PBS and cover-slipped. Images were then visualised using structured illumination microscopy (SIM).

### 2.9. Analysis of Real-Time Extracellular Oxygen Consumption (OCR)

PA-driven oxygen consumption was analysed using an extracellular oxidation assay kit (Abcam, Cambridge, UK—ab197243). Exponentially growing cultured hCMEC/D3s (6 × 10^4^ cells per well) were seeded in 96-well plates and incubated overnight in 150 μL of media in 5% CO_2_ at 37 °C. Cells were then rinsed twice with 37 °C prewarmed fatty acid (FA)-free medium (base measurement medium); then, 150 μL of PA (range 10, 50, 100, 200 and 400 μM—final concentration), 10 μL of extracellular oxygen consumption reagent and FA measurement medium were added to each well without cells and used as the signal control. An additional 1 μL (0.625 μM) of the fatty acid oxidation (FAO) activator, FCCP (carbonyl cyanide-4-(trifluormethoxy) phenylhydrazone, Abcam—ab120081), was subsequently supplemented into the wells as a positive control, or 1 μL (2.5 μM—final concentration) of antimycin A (Abcam, Cambridge, UK—ab141904) as a negative control of cellular respiration. Each well was sealed with 100 μL of 37 °C prewarmed, high-sensitivity mineral oil (Abcam, Cambridge, UK—ab197243), taking care to avoid bubbles. Plates were immediately read at excitation/emission wavelengths of 380 nm/650 nm at 1.5 min intervals for 30 min at 37 °C in a SpectraMax M2 fluorescence microplate reader (Molecular Devices, San Jose, CA, USA).

### 2.10. Image Analysis

The image co-localisation analysis was conducted using IMARIS 9.9 software (Oxford Instruments, Abdingdon, UK), and Mander’s coefficient analysis and regression tests were applied [27]. Briefly, to normalise the images across treatments, the images were thresholded by selecting a baseline where the red and green channels were most separated. Specifically, the separate tracks’ thresholds were set to just above their background levels (for an example, see Supplementary Figure S3). Cytosolic pixel intensity analysis was administered by selecting regions of interest on cells’ cytosol and extracting pixel frequency and intensity information. The intensity information was then normalised between 0 and 1 to enable comparisons between treatments and plotted as a histogram (for an example of how the cytosol ROI was selected, see Supplementary Figure S4).

### 2.11. Statistical Analysis

Statistical ANOVA was performed with Prism 9.4 software (GraphPad, San Diego, CA, USA). Test, group sizes and *p*-values are provided in the corresponding figure legends. The *p*-values < 0.05 were considered statistically significant. Linear regression models (LM) were fitted with Rv4.01. For data that indicated a non-normal residual relationship, a nonlinear term was included in the model, i.e., a polynomial term.

### 2.12. Data Availability

The dataset supporting the conclusion of this article is included within the main article and in the Supplementary Materials. No data generated in this study were downloaded into a public domain.

## 3. Results

### 3.1. Identification of Areas and Characterisation of CNS Tissue Samples

**Figure 1:**
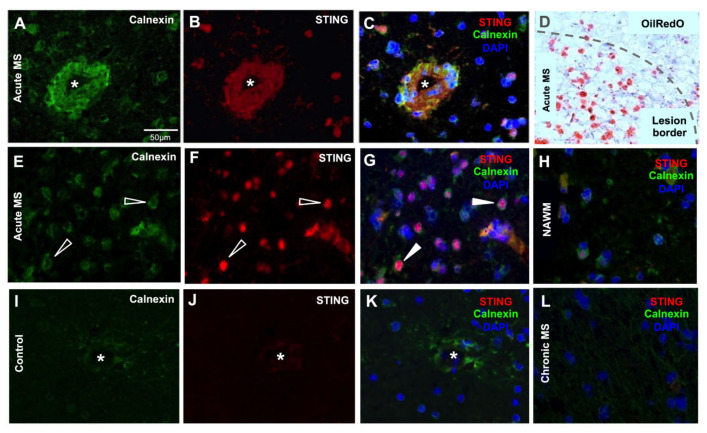
Representative immunofluorescent labelling of STING in acute, chronic, NAWM MS brain tissue and control WM. (**A**–**C**) Increased levels of STING were present in the endothelial cells, which were heavily stained for calnexin, of brain microvasculature, marked with an asterisk in the acute MS lesion. (**D**) Lesions were assessed with the lipid oil-red-O staining adjacent to immune-stained sections. (**E**,**F**) STING-positive neuronal cell bodies (open arrowhead) were seen in the proximity of the subcortical white/grey matter border in the acute MS lesion (filled arrowhead in (**G**)), whilst MS NAWM (**H**), chronic (**L**) and non-MS control tissues (**I**–**K**) demonstrated much lower STING immunopositivity and a lack of co-localisation with the calnexin-positive structures. Both the chronic MS lesions and NAWM from the acute MS brain demonstrated low levels of STING immunosignals in endothelial cells. Scale bar represents 50 μm.

Standard stains were used to find regions of interest in both pathological and control tissue sections. For the MS tissue samples, oil-red-O staining of myelin was used to identify demyelinating areas of white matter. In normal control tissue, the white matter oil-red-O staining produced an even, pink stain of myelinated tissue matrix. A similar staining pattern was seen in non-lesioned MS white matter (normal appearing white matter-(NAWM)). In contrast, MS lesions were classified as acute or chronic based on the presence of oil-red-O lipid-filled macrophages and the amount of demyelination and haematoxylin-stained cellularity at the lesion border. An acute MS lesion consisted of many lipid-filled macrophages, demonstrating an active area of demyelination of axonal fibres and a defined border (Figure 1D). However, in a chronic lesion, no lipid-filled macrophages were present, and the lesion area was completely demyelinated [28]. The acute lesion borders were marked on the adjacent sections of the very same sample and used for subsequent immunofluorescent staining. In previous murine and human studies, we have demonstrated that the protein level of calnexin is raised in brain endothelial cells within inflammatory regions of the CNS compared to healthy tissue [29,30] (Supplementary Figure S1). Subsequently, calnexin acts as a good marker of inflamed endothelial cells. An anti-calnexin antibody staining against calnexin was used as a guide for detecting inflamed capillaries (Figure 1A & Supplementary Figure S1), and the tissue co-stained for STING (Figure 1B & Supplementary Figure S2). As shown in the Figure 1C overlay and Figure S2, both calnexin and STING were particularly abundant in blood capillaries from within acute/chronic lesions of white matter of MS brain tissue compared to control brain tissue (Figure 1I–K).

**Figure 2:**
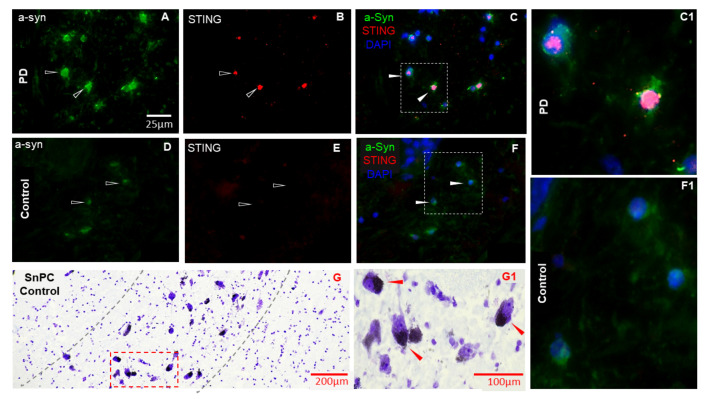
Immunofluorescent staining of representative tissue sections in PD and control SnPC. An increased STING immunosignal was present in highly α-synuclein-positive cell bodies in the PD brain (open arrowhead in (**A**,**B**), respectively) and demonstrated strong co-localisation (filled arrowhead in (**C**), enlarged in (**C1**)), while the control tissue (**D**–**F**) showed a minimal STING signal (**E**) with little α-synuclein present (**D**), and thus, a lack of co-localisation (filled arrowhead in (**F**), enlarged in (**F1**)). SnPC was identified using Nissl-stained midbrain sections (**G**), based on the presence of neuromelanin containing large (~50 μm) somatic profiles, as putative dopaminergic neurons (red arrowheads in (**G1**)) in both the non-neurodegenerative control tissue (**G1**) and PD brain samples. Scale bar for (**A**) is representative for (**A**–**F**).

**Figure 3:**
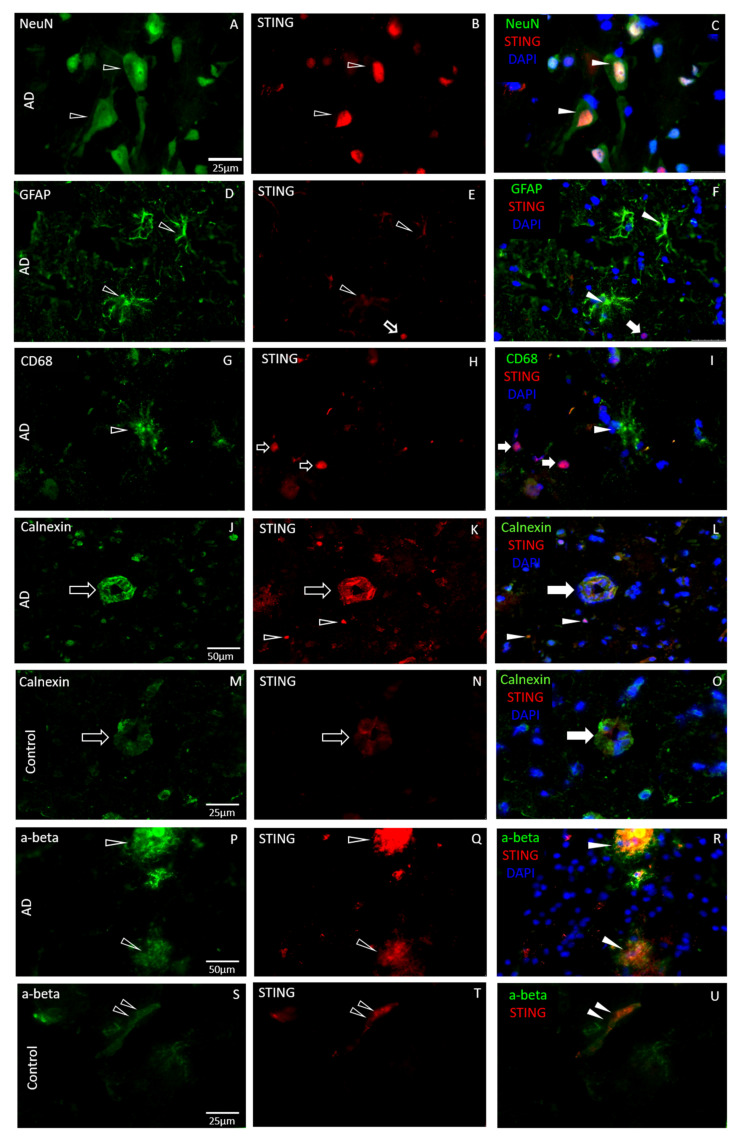
STING immunostaining in neural cells in association with amyloid-β in AD and control brain parenchyma. NeuN-labelled putative pyramidal cells (open arrowhead in (**A**)) show an intense STING immune signal (**B**,**C**), while astrocytes (GFAP) (**D**,**F**) and microglia (CD68) (**G**,**I**) demonstrated weak STING positivity (open arrowhead in (**E**,**H**), respectively). NeuN-positive cells contain high levels of STING (filled arrow in (**C**)) compared to the GFAP-expressing astrocytes and CD68-positive myeloid cells (filled arrowhead in (**F**,**I**), respectively) that demonstrate reduced STING positivity. Increased STING positivity was present in the endothelial cells (open arrow in (**J**,**K**) and filled arrow in (**L**)) in the frontal-temporal cortex of the AD brain, in contrast to blood vessels of the control tissue with decreased STING levels (open arrow in (**M**,**N**) and filled arrow in (**O**)). Cortical amyloid-β depositions demonstrated a robust STING presence and strong co-localisation with the pathological plaques (open arrowheads in (**P**,**Q**) and filled arrowheads in (**R**)). Only a low level of STING was detected in amyloid-β depositions, found occasionally in the brain microvasculature of the age-matched control cortex (double open arrowheads in (**S**,**T**)), with no obvious abnormal amyloid pathology in the same structure (double filled arrowheads in (**U**)). Neuronal cell bodies were strongly labelled with STING in the AD brain (open arrows in (**H**) and arrowheads in (**K**)) and co-localised with DAPI (filled arrows in (**I**) and arrowheads in (**L**)).

**Figure 4:**
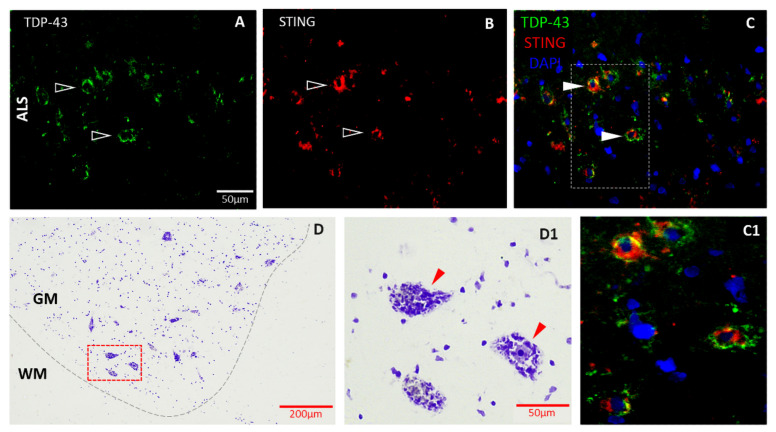
Representative examples of immunohistopathological examination of STING in motor neurons. Tissue samples from the lower cervical segment of the spinal cord of a sporadic ALS patient ((**A**–**C**) and (**C1**)). STING showed co-localisation with TDP-43 positive large cell bodies (filled arrowhead in (**C**), enlarged in (**C1**)) of presumed motor neurons. Adjacent to immunolabelled sections, Nissl contrast staining was used for identifying motor neurons (arrowhead in (**D1**)) in both control (**D**,**D1**) and ALS spinal cord samples. GM: grey matter, WM: white matter.

The SnPC of the Parkinsonian and control midbrain was identified based on the presence of large (~50 μm) neuromelanin-containing bodies in Nissl-stained sections (Figure 2G(G1)). Consecutive tissue sections were selected for the triple immunolabelling, where cellular profiles of putative dopaminergic neurons were identified in non-neurodegenerative controls and their lesions in the diseased brain tissue samples, respectively. In the frontal temporal cortex of AD and corresponding control brains, amyloid-β immunostaining was used to characterise deposits of misfolded proteins (Figure 3P,R). Lower cervical sections selected from the ALS spinal cord and the corresponding control tissues were contrasted with Cresyl violet (Figure 4D(D1)) to identify and locate motor neurons in the grey matter of the ventral horn and guide the orientation and selection of subsequent tissue sections to be probed by specific antibodies against STING and toxic protein aggregates (TDP-43). The putative cell bodies of motor neurons deriving form ALS patients’ tissue samples demonstrated accumulation of pathological aggregates of the toxic protein TDP-43 (Figure 4A) following immunofluorescent detection. The same section was subsequently probed for STING (Figure 4B). The STING immunosignal in the defined neurons showed co-localisation with misfolded protein (TDP-43) in the cellular bodies (Figure 4C, enlarged in (C1)).

### 3.2. STING Expression Is Elevated in Neurodegenerative Diseases in Endothelial and Neural Cells

We found that STING is predominantly expressed in endothelial cells of brain capillaries and neuronal cell bodies of the pathological brain tissue samples. In the acute subcortical white matter of the MS brain, there was evidence of an abundant STING presence in cell bodies (Figure 1). High levels of STING immunostaining showed co-localisation with putative pyramidal cells in grey matter of the frontotemporal cortex of AD patients (Figure 3), in the SnPC of PD patients (Figure 2) and in putative motor neurons of the ALS spinal cord sections (Figure 4). Activated microglia and astrocytes also demonstrated elevated STING in the cerebral cortex of AD patients of both familial and sporadic cases (Figure 3D–I), but only in moderation. In general, the intensity of STING staining in these former cells was less prominent compared to that of neurons (Figure 3A–C) and endothelial cells (Figure 3J–L). Misfolded protein plaques and neurons containing protein aggregates showed high levels of the STING immunofluorescent signal (Figure 3P–R). In contrast, STING expression in the control tissues was much less evident in all cell types in specific regions associated with of all four neurodegenerative diseases. In this study, we have demonstrated that neurons, which contain protein aggregates in multiple proteopathies (PD, AD, ALS) and the neuroinflammatory condition MS, are highly STING-immuno-positive (Figure 1, Figure 2, Figure 3 and Figure 4), and are often localised in association with activated microglia, astrocytes and endothelial cells (Figure 3). In contrast, equivalent regions of the non-neurodegenerative control CNS tissue showed quiescent STING immunosignals (Figure 1, Figure 2 and Figure 3). Here, we have provided the first demonstration that four main neurodegenerative diseases have increased STING in specific cell types within different CNS areas associated with the pathology.

We observed that STING-immuno-positive neuronal cell bodies were prevalent within the region of the SnPC of the PD brain tissue and demonstrated co-localisation with putative dopaminergic neurons possessing intense α-synuclein staining (Figure 2). The SnPC region was confirmed by the presence of neuromelanin in the neuronal cell bodies in Nissl-stained sections (Figure 2G(G1)). Only a few highly STING-positive neuronal cell bodies were observed in the associated brain region of the age-matched control tissue sections (Figure 2D–F1), in contrast to the PD midbrains, where STING was highly prevalent and demonstrated strong co-localisation with α-synuclein (Figure 2A–C1). Frontal-temporal cortical sections from AD patients were used for double immunostaining, and localisation of STING was confirmed in distinct neural cell types in the brain tissue. A high-intensity STING immunosignal was obvious in presumed NeuN-positive neuronal cell bodies (Figure 3A–C) and brain endothelial cells identified by morphological features of cross-sections of the brain vasculature in the cortical regions used for the histopathological examination (Figure 3J–L). Cell-specific markers for distinguishing glial cell subtypes, astrocytes (GFAP) and microglia (CD68) (also labelled monocytes and macrophages), were double-stained with STING to assess their localisation in various CNS cell types (Figure 3D–I). Both glial cell populations showed low-intensity STING signals, suggesting lower STING activity in these cell types compared to neurons or brain endothelial cells. Interestingly, in grey matter, intense GFAP staining was seen in the pathological tissues, suggesting that activated astrocytic processes are often associated with intense STING-positive neuronal cells in cortical parenchyma (Figure 3D–F).

### 3.3. Disruption of Mitochondrial Respiration by Palmitic Acid

**Figure 5:**
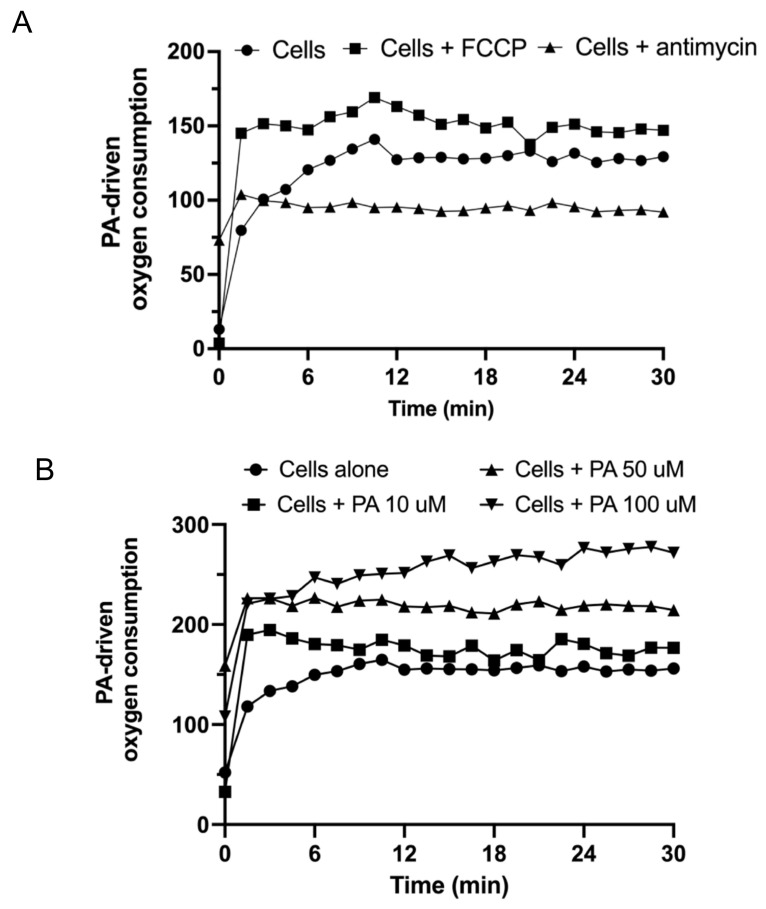
Increased oxygen consumption in PA-treated hCMEC/D3s. (**A**) Representative traces of OCR of hCMEC/D3s in the presence or absence of FCCP or antimycin. (**B**) Dose-dependent increase in hCMEC/D3s OCR with exposure to increasing concentrations of PA. Extracellular oxygen consumption signal was measured at 1.5 min intervals for 30 min at Ex/Em = 380/650 nm.

An observed consequence of neurodegeneration is the presence of damaged mitochondria, due to leakage of electrons from the electron transport chain (ETC) that interact with molecular oxygen and generate reactive oxygen species (ROS) [31,32]. These free radicals can damage mitochondria, leading to release and damage of mitochondrial DNA, which is 100 times more susceptible to damage than nuclear DNA [33]. Such DNA could act as an activator of the cGAS-STING pathway. Therefore, we measured the oxygen consumption of mitochondria in hCMEC/D3s treated with and without PA, a known inducer of ROS. The electron transport chain uncoupler, FCCP, was used to induce maximal ETC activity. FCCP is a potent uncoupler of mitochondrial oxidative phosphorylation. Actimycin A was used as a complex III inhibitor and a negative control of respiration (Figure 5A). Antimycin A induced a rapid loss in mitochondrial membrane potential, and a collapse of oxidative phosphorylation. Palmitic acid treatment reduced mitochondrial function by increasing oxygen consumption rates in hCMEC/D3s proportionally with the PA concentration (10–100 μM) (Figure 5B). Mitochondrial oxidative function is required to transform the energy of nutrients into a proton gradient pump used to make ATP via the ETC. The real-time OCR was increased in the presence of PA compared to basal respiration and appeared to supersede the maximal respiratory capacity observed from the oxygen consumption after FCCP stimulation, which is known to be a strong indicator of mitochondrial energetic dysfunction [34].

### 3.4. Palmitic Acid Caused dsDNA Leakage into the Cytosol of hCMEC/D3s

**Figure 6:**
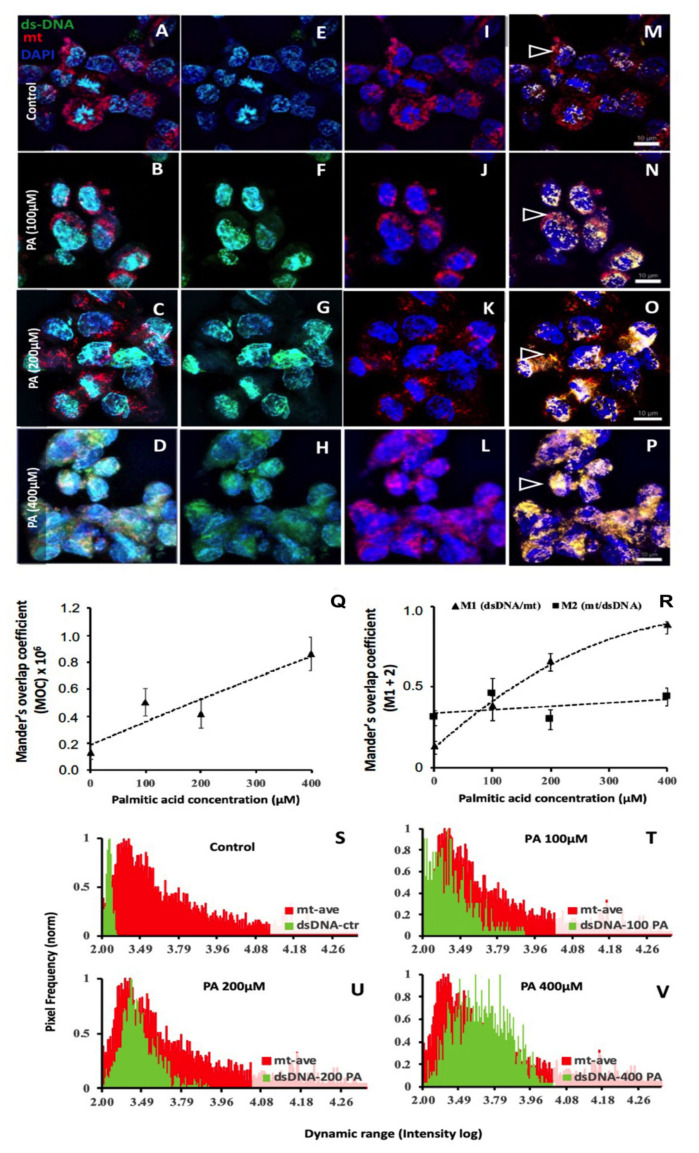
Mitochondrial DNA release and co-localisation analysis of mitochondria (mt) and dsDNA in PA-treated hCMEC/D3s. (**A**–**D**) Far-left column—Representative images of hCMEC/D3s stained with the dsDNA marker PicoGreen, DAPI (blue) and the mt-specific marker Mito-Tracker Red. (**E**–**H**) Centre-left column—hCMEC/D3 cells showing tracks with the dsDNA marker PicoGreen and DAPI (blue). (**I**–**L**) Centre-right column—hCMEC/D3 cells showing tracks labelled with DAPI (blue) and Mito-Tracker Red. (**M**–**P**) Far-right column—hCMEC/D3 cells showing leakage of dsDNA (depicted by yellow pixels), which increases in the cell’s cytosol with the increasing PA concentration. Open arrowheads (**M**–**P**) indicate examples where the increase in dsDNA in the cytosol is apparent, and thus co-localisation (yellow area) between dsDNA and mt is evident. (**Q**) The positive correlation of the increasing co-occurrence between mt and dsDNA, across the PA treatment. (**R**) Coefficient M1 (dsDNA/mt) indicates that the fraction of dsDNA that overlaps with mt increased with the PA concentration, revealing a clear dose-dependent relationship (*n* = 6–8). Coefficient M2 (mt/dsDNA) demonstrates that the fraction of mt overlapping with dsDNA did not change across PA concentrations (*n* = 6–8). (**S**–**V**) Cytosolic pixel intensity analysis showed a shift of the ‘green’ peak to the right, supporting that dsDNA increased with the PA concentration. Pixel profiles were extracted from where the open arrowheads are indicated (**M**–**P**). Pixel frequency is normalised between 0 and 1, and mt represents a logged average from across the PA treatments. Scale bar shown on immunofluorescent images represents 10 μm for images (**A**–**P**).

To determine if PA induces DNA release from stressed hCMEC/D3s, we used PicoGreen as a marker of dsDNA, which is known to visualise dsDNA in non-permeabilised cells [26]. Our results showed that hCMEC/D3s treated with concentrations of PA (100–400 μM) caused DNA leakage into the cytosol of the cells (Figure 6). In untreated hCMEC/D3s, the intense blue fluorescence staining of nuclear DAPI can be seen coinhabiting the nucleus with the PicoGreen (dsDNA). There is very little evidence of dsDNA staining in the cytosol of untreated cells (Figure 6E). Palmitic acid is known to trigger nuclear [35] and mitochondrial [36] DNA damage. Leakage of such DNA into the cell’s cytosol is known to activate cGAS [37,38].

Thresholded quantitative co-localisation analysis revealed a positive PA concentration-dependent co-occurrence between PicoGreen (dsDNA) and Mito-Tracker Red (mt) in Figure 6. Expressly, a plot of the simple spatial overlap of the dsDNA and mt probes (represented by overlapping pixels) indicated a significant positive relationship between the pixels’ co-occurrence (dsDNA = mt) and the concentration of PA administered (a regression test, *p* < 0.05; Figure 6Q). A regression test of Mander’s coefficient M1 (the fraction of signal overlap between dsDNA (green) and mt (red)) showed a significant increase, corresponding to the concentrations of PA that the cells were exposed to (regression test, *p* < 0.05, Figure 6R). This supports the notion that an increase in cytosolic dsDNA is associated with PA treatment. In contrast, a regression test of Mander’s coefficient M2 (the fraction of signal overlap between mt (red) and dsDNA (green)) did not demonstrate a significant relationship with the PA concentration (*p* > 0.05) (Figure 6R). These data suggest that the amount of mt that relates to dsDNA did not change as would be expected, as the proportion of mt in a cell should not have been affected by the PA concentration (see details in Supplementary Tables S3–S6). In addition, cytosolic pixel intensity analysis also demonstrated that in the hCMEC/D3s cells, dsDNA increased with the PA concentration, as shown in Figure 6S–V.

### 3.5. Palmitic Acid and cGAS Activate the STING-TBK1 Pathway and Induce hCMEC/D3 Inflammation

**Figure 7:**
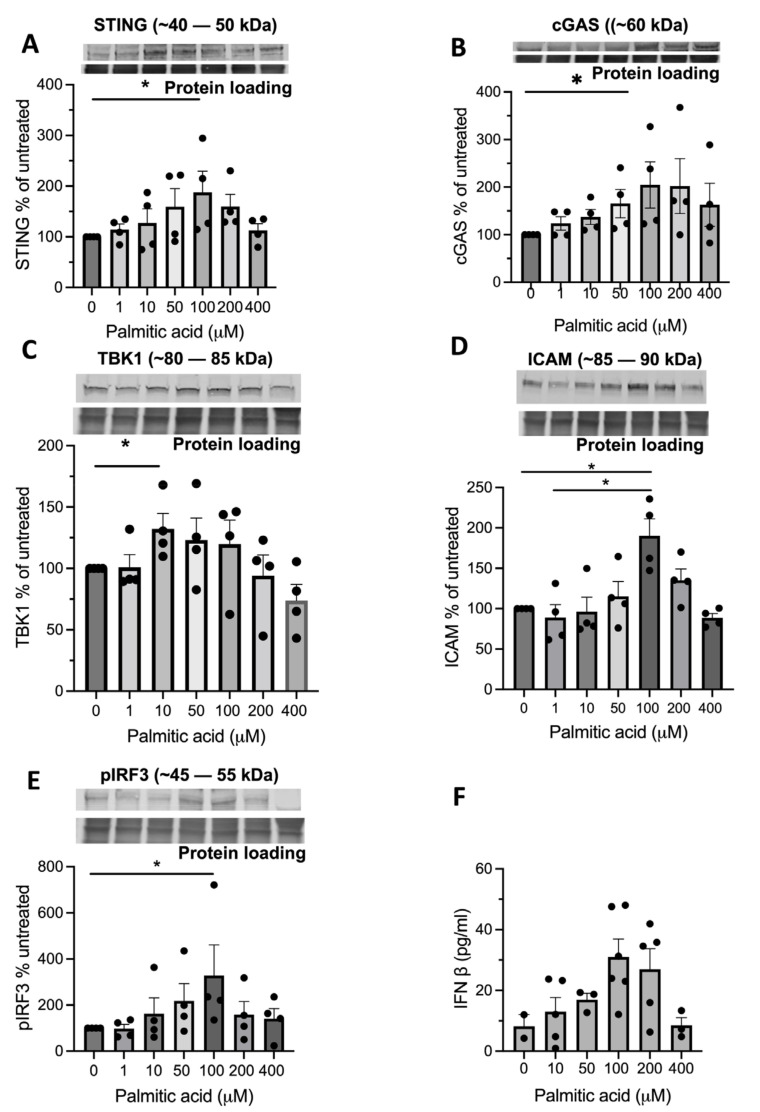
Effect of PA treatment on cGAS-STING signalling and downstream ICAM and IFN-β production in hCMEC/D3s. (**A**,**B**) Effect of palmitic acid treatment on cGAS and STING. Dose response effect of PA on (**C**) TBK-1, (**D**) pIRF3 and (**E**) ICAM production, *n* = 5–6 experiments/condition. (**F**) Release of IFN-β from cells, *n* = 5 replicates. Data are expressed as mean% change ± SEM from untreated cells. Mann–Whitney test, * denotes *p*-values < 0.05. Dots denote individual subject values.

Having observed STING levels in the CNS microvascular endothelial cells of patients with different NDs, we wished to determine if the metabolic stress-induced fatty acid PA altered protein levels downstream in the STING signalling pathway in hCMEC/D3s. PA is known to be elevated in the brain of patients with Parkinson’s [39] and Alzheimer’s diseases [40] and the serum of MS [41] and blood cells of ALS patients [42]. We treated hCMEC/D3s with PA at concentrations ranging from 1 to 400 μM. We observed that 100 μM of PA had a significant effect of increasing the levels of STING in hCMEC/D3s (Figure 7A), and 50 μM of PA significantly increased the cGAS expression (Figure 7B). The higher treatment doses of PA (200–400 μM) had minimal effect on STING and cGAS protein levels. Increased expression of ICAM on brain endothelial cells was observed in fresh-frozen post-mortem tissue from patients with neuroinflammatory diseases [43,44]. As the downstream signalling molecule TBK-1 and the transcription factor pIRF3 of the STING pathway are in part known to bind to the ICAM gene promoter and induce ICAM expression on aortic endothelial cells, leading to increased cell attachment [36], we further investigated whether PA increased these downstream signalling molecules, and subsequently, ICAM expression and IFN-β on hCMEC/D3s. Here, we observed a dose-dependent response to PA, which resulted in an increase in TBK-1, pIRF3, ICAM expression and the IFN-β level in hCMEC/D3s (Figure 7C–F).

### 3.6. Activation of Type I IFN Expression in hCMEC/D3s

To investigate the innate immunity of hCMEC/D3s in response to PA-induced mitochondrial stress and STING activation, the capability of IFN-β release from cells was examined under specific agonist stimulation with PA. Saturated fatty acids such as PA have been implicated in inducing STING in endothelial cells [36], and STING, in turn, can activate type I IFN responses [45], but the direct effect of PA on IFN-β in brain endothelia is unclear. As shown in Figure 7F, after treatment with PA, hCMEC/D3s released IFN-β in a dose-dependent fashion. The reduced detection of IFN-β in the media of cells treated with 400 μM of PA may reflect the reduced cell viability after such treatment.

## 4. Discussion

In this study, we have observed increased expression of STING in the CNS microvasculature and neuronal cells of individuals with various NDs. We showed that elevated levels of STING were in close proximity to the build-up of protein aggregates such as TDP-43 in ALS, α-synuclein in PD and amyloid-β plaques in AD in a variety of neural cell types. The role of the cGAS-STING pathway in the human brain is only beginning to be explored in terms of a protective or pathological consequence. Recent evidence has revealed that STING signalling drives neuroinflammation in traumatic brain injury [46]. A downstream implication of STING activation is the production of type I IFNs. Elevated levels of type I IFNs have been found in brain post-mortem tissues of human AD [15], PD [47] and in ALS myeloid cells [48]. Further evidence that STING plays a role in neuroinflammation arises from STING knockout models, which have shown that elimination of STING alleviates some disease pathology in PD mice models [49]. What was striking was the particularly strong STING expression observed in endothelial cells in disease tissues compared to the age-matched non-neurodegenerative control CNS tissue. STING expression is known to occur in both normal and diseased tissue and may be important in anti-tumour development. Endothelial cells are the main producers of type I IFNs [50], which raises the questions, are the CNS endothelia producing type I IFNs in response to STING activation, and what is eliciting the increased STING production?

It was of interest to observe the co-localisation of pathological protein aggregates near elevated STING expression in neural cells (Figure 2, Figure 3 and Figure 4), which may reflect ER and mitochondrial stress on the cells induced by toxic protein build-up. In some other diseases, such as arterial hypertension ER, mitochondrial stress and accumulation of protein aggregates have all been implicated in the activation of STING [51]. In PD, overexpressed STING has been seen in the SnPC of patients, and might be important in PD pathogenesis [52]. In our human CNS tissues, we did not monitor IFN levels in these tissues, so we do not know if interferons were subsequently generated. However, Gonzalez-Riano et al., using a metabolomics approach to find PD biomarkers in a large Spanish population, found that PA levels were reduced in subjects who went on to develop Parkinson’s disease and proposed that alpha synuclein acted as a transporter, promoting uptake of PA into the brain where it could promote damage [53]. In the AD tissue, STING was predominantly detected in NeuN-positive cortical neurons and putative pyramidal cells, but it was less evident in GFAP-positive astrocytes or in CD68-positive microglia in AD tissue. This is despite a recent transgenic mouse model of AD reporting that enhancement of the cGAMP-STING pathway leads to the upregulation of microglia TREM2, which in turn suppresses amyloid-β-related neuropathology [54].

In the in vitro arm of this study, PA was used as an agonist of neuroinflammation, since PA and other saturated free fatty acids are associated with neurodegenerative processes [55]. In our study, PA treatment of hCMEC/D3s led to the escape of dsDNA into the cytosol of cells (Figure 6E–H), which is known to act as a ligand for the activation of the DNA sensor cGAS [2]. The aim was not to address whether the detected cytosolic DNA originates from damaged mitochondria or nuclear DNA. However, analysis of the DAPI staining indicated that the nuclei staining was consistent across treatments, indicating unlikely leakage of dsDNA from the nucleus. Others have investigated this in human aortic endothelial cells (HAECs), in which the source of the DNA appeared to be predominantly mitochondrial [36,56,57]. Subsequent STING activation was associated with an increased TBK-1 production (Figure 7C) and more pIRF3 (Figure 7E). Once pIRF3 enters the cell nucleus, it can bind to various gene promoters and enhance the expression of a number of downstream inflammatory factors in the cells (e.g., IFN-β and ICAM-1). We detected that PA-activation of hCMEC/D3s led to increased ICAM expression on the cell surface, confirming the association between pIRF3 and ICAM expression, recently observed by Mao and colleagues [36]. We also observed that PA-treated endothelial cells released IFN-β in a dose-dependent manner. Interestingly, endothelial cells in the presence of IFN-β become less permeable and increase the levels of tight-junction proteins [58]. It remains to be investigated if the release of IFN-β by endothelial cells is a means to stabilise the BBB upon STING activation. STING signalling is associated with worsened neurodegenerative disease in mice [49,59,60], and our histology studies in human subjects (Figure 1, Figure 2, Figure 3 and Figure 4) appear to confirm these animal observations.

**Figure 8:**
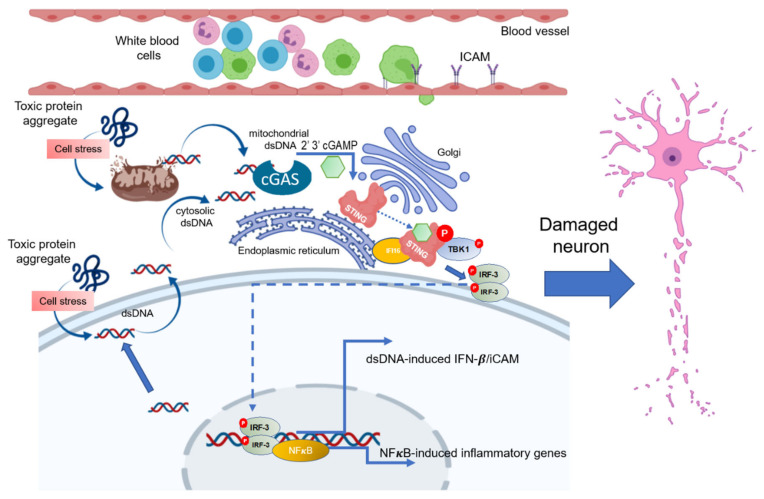
Schematic diagram of activation of the cGAS-STING pathway in the CNS. Cytosolic DNA is recognised by cGAS, leading to formation of a cGAS-DNA complex. This leads to synthesis of a 2′ 3′-cGAMP–STING ligand. Activated STING translocates from the ER to the proximity of the Golgi, where STING is post-translationally modified (palmitoylated and phosphorylated) by TBK-1. This provokes STING to recruit IRF3, leading to IRF3 phosphorylation and dimerization, and the pIRF dimer travels to the nucleus to drive expression of both type I interferons and ICAM expression, as well as NFκ-B inflammatory genes. This diagram was created with BioRender.com.

Our detection of STING activation in the brain endothelial cells of neurogenerative patients may also be a marker of type I IFN release, notably IFN-β. While IFN-β therapies are of benefit to a cohort of MS patients, some MS patients treated with IFN-β have been observed to go on to develop PD-like symptoms [61]. Recent studies have shown that the IFN pathway is grossly upregulated in clinical AD [62] and PD [47]. Although IFNs are well-known for their anti-viral properties, they can exert anti-inflammatory effects [63]. An increase in STING-mediated IFN-β expressed by BBB endothelial cells could indicate a reduction in immune cell trafficking or an attempt to retain BBB integrity between the blood and the CNS, and not necessarily neuroinflammation [64]. However, somewhat paradoxically, in earlier studies, IFN-β was shown in murine models of experimental autoimmune encephalomyelitis (EAE) to decrease the abundance of adhesion molecules on brain capillaries such as ICAM-1 and VCAM-1 [65]. In our study, we saw an increase in ICAM and IFN-β expression of hCMEC/D3s treated with PA, possibly via IRF3 phosphorylation and nuclear translocation of this transcriptional inducer of IFN-β and ICAM. A previous study [36] showed that PA-treated endothelial cells led to ICAM-induced expression via the STING-pIRF3 pathway. Overall, in neurodegeneration, activation of the cGAS-STING pathway could be through viral DNA but, could also be through self-DNA (nuclear or mitochondrial) triggered by pathological protein fibrils (Figure 8) or lipid toxicity. Viral aetiologies for NDs have received a lot of attention, and these data do not rule out viral DNA as activating the cGAS-STING pathway in NDs. However, our data in general support a self-DNA story (cellular stress and DNA release caused by build-up of fibrillar proteins), or from PA—metabolic activation of human brain endothelial cells.

## 5. Conclusions

Several NDs lead to neural cell damage and a reduced ability to remove damaged mitochondria in response to stress, resulting in STING-mediated inflammation. Our observations suggested that both the brain microvasculature and neurons in AD, PD, ALS and MS demonstrated a common increase in cGAS-STING pathway signalling compared to the age-matched control tissue. We have demonstrated that nuclear or mitochondrial DNA escape induced by PA-mediated inflammation is recognised by cGAS, leading to activation of the STING-TBK-1 signalling pathway and increased expression of important inflammatory factors.

## Data Availability

All data generated or analysed during this study are included in this published article and its Supplementary Materials.

## Supplementary Materials

The following supporting information can be downloaded at: https://www.mdpi.com/article/10.3390/biomedicines11051375/s1, Figure S1: Abundant expression of calnexin in an acute lesional MS blood vessel. Figure S2: Co-staining of calnexin and STING in a cross section and longitudinal section of a blood capillary. Figure S3: Examples of baseline thresholds set for colocalization analysis. Figure S4: Semi-quantification of pixel analysis and cytosolic dsDNA/Mitochondria proximity. Table S1: Patient tissue details. Table S2: Primary and secondary antibodies used for immunohistochemistry and immunoblotting. Table S3: The coefficients generated from thresholded quantitative colocalization analysis using IMARIS 9.9. Table S4: Regression analysis of the overlapping colocalized pixels (MOC). Table S5: Regression analysis of the Manders coefficient A (M1) of co-occurrence. Table S6: Regression analysis of the Manders coefficient A (M2) of co-occurrence.

## Abbreviations

AD = Alzheimer’s disease; ALS = amyotrophic lateral sclerosis; Ctx = cortex; FTCx = frontal-temporal cortex; MS = multiple sclerosis; PD = Parkinson’s disease; PMD = post-mortem time; SnPC = substantia nigra pars compacta; WM = white matter; NAWM = normal appearing white matter; PA = palmitic acid; cGAS = cyclic GMP-AMP synthase; STING = stimulatory interferon gene; TBK-1 = TANK-binding kinase 1; ICAM 1 = intracellular adhesion molecule 1; pIRF3 = phosphorylated interferon regulatory factor 3; IFN-β = interferon β; mt = mitochondria; NDs = neurodegenerative diseases; ETC = electron transport chain; ROS = reactive oxygen species; BBB = blood–brain barrier; IFNs = interferons; hCMEC/D3 = human cortical micro-vessels endothelial cells/D3; IFI16 = Interferon-gamma-induced protein 16; Aβ = β-amyloid protein; BSA = bovine serum albumin; TDP-43 = TAR DNA-binding protein 43; FA = fatty acid; FAO = fatty acid oxidation; FCCP = carbonyl cyanide-4-(trifluormethoxy) phenylhydrazone; LM = linear regression models; LFN = lane normalisation factor; SIM = structured illumination microscopy; OCR = real-time extracellular oxygen consumption.
