# Supplementary material for: STING-Triggered CNS Inflammation in Human Neurodegenerative Diseases
Source: Biomedicines. 2023 May 5;11(5):1375. doi: 10.3390/biomedicines11051375 (PMC10216406; doi:10.3390/biomedicines11051375)
Supplement: Supplementary file 1 [file biomedicines-11-01375-s001.zip › biomedicines-2305981-supplementary.pdf]

Supplementary Material

Supplementary Table S1: Patient tissue details

Pathology	Age (y)	Sex	PMD (hrs)	Disease type/Cause of death	CNS region	Disease level	No of Sections analysed (*n*)
**AD**	80	M	15	AD with amyloid-β deposition	FTCx	Braak VI	4
	80	F	13	AD with mild amyloid-β angiopathy	FTCx	Braak V	4
	81	F	21	AD with focal amyloid-β angiopathy and localised limbic TDP-43 pathology	FTCx	Braak VI	4
	72	F	3	AD Familial (APP717 (VAL-ILE)) V717LAPP	FTCx	NA	4
	59	F	89	NA	FTCx	Braak VI	4
	42	M	6	AD Familial (PS1 (DELTA 4)	FTCx	NA	4
**AD Controls**	81	M	18	Cerebral infarct Normal brain with no obvious abnormal Tau and amyloid-β pathology	FTCx	Braak I	2
	72	F	10	NA	FTCx	NA	4
	87	F	22	Normal brain with no obvious abnormal Tau and amyloid-β pathology	FTCx	NA	3
	92	F	17	Some Tau deposition	FTCx	NA	4
**PD**	81	M	37	NA	SnPC	Visible lesion in SnPC	2
	64	M	45	NA	SnPC	Visible lesion in SnPC	3
	80	F	57	NA	SnPC	Visible lesion in SnPC	2
**SnPC Controls**	89	M	38	NA	SnPC	No obvious lesion in SnPC	4
	80	F	29	NA	SnPC	No obvious lesion in SnPC	2
	87	F	51	NA	SnPC	No obvious lesion in SnPC	3
**MS**	56	F	13	Sepsis and MS	Ctx, WM	NAWM	4
	56	F	13	Sepsis and MS	Ctx, WM	Acute, Secondary progressive	4
	35	F	9	MS	Ctx, WM	Acute, Secondary progressive	3
	60	F	24	NA	Ctx, WM	Chronic MS	4
**MS Controls**	64	M	18	Cardiac failure	Ctx,WM		4
	82	M	21	NA	Ctx, WM		4
**ALS**	70	M	40	Sporadic ALS (1y3months diagnosis to death)	Lower cervical (Sc)	Sclerotic appearance, significant shrinkage in tissue volume	3
	79	F	14	Sporadic ALS (5y from diagnosis to death)	Lower cervical (Sc)	Sclerotic appearance, slight shrinkage in tissue volume	4

	65	F	30	Sporadic ALS (1y11month diagnosis to death)	Lower cervical (Sc)	Sclerotic appearance, shrinkage in tissue volume	4
**ALS Controls**	68	M	40	Heart disease	Lower cervical (Sc)	Normal appearance	4
	80	F	22	Pulmonary embolism	Lower cervical (Sc)	Normal appearance	3

Abbreviations used: AD; Alzheimer's disease, ALS; Amyotrophic lateral sclerosis, PD; Parkinson's disease, MS; Multiple Sclerosis, PMD; Postmortem time, FTCx; Frontal-temporal cortex, SC; Spinal cord, SnPC; Substantia Nigra Pars Compacta, Ctx; Cortex, WM; White matter; NAWM, normal appearing white matter; NA, not available

Supplementary Table S2: Primary and secondary antibodies used for immunohistochemistry and immunoblotting

Type	Origin	Clone	Target	Dilution	Source
			**Primary antibodies**		
			HISTOLOGY		
Anti-STING	Mouse	MC	Activated cGAS/STING pathway in neuronal cell types	1:100	R&Dsystems MAB7169
Anti-TDP43	Rabbit	MC	Degenerating motor neurons in the spinal cord	1:100	Abcam (Ab109535)
Anti-amyloid β	Rabbit	PC	Misfolded protein, amyloid-β deposition	1:100	Abcam (Ab2539)
Anti-calnexin	Rabbit	PC	Binds to partially folded or misfolded proteins ensuring proper folding of proteins	1:100	Enzo ADI-SPA-860-F
Anti-α synuclein	Rabbit	MC	Misfolded protein, synuclein build up in the neurons	1:100	Abcam (Ab209538)
Anti-GFAP	Rabbit	PC	Activated Astroglia	1:100	ThermoFisher (PA5-16291)
Anti-CD68	Rabbit	PC	Activated myeloid cells (monocytes, macrophages, microglia)	1:100	ThermoFisher (PA5-32330)
Anti-NeuN	Rabbit	MC	Neuron selective marker in the cortex	1:100	Abcam (Ab177487)
			IMMUNOBLOTTING		
Anti-STING	Mouse		Activated cGAS/STING pathway in neuronal cell types	1:100	R&Dsystems MAB7169
Anti-phospho-IRF3 (Ser396) (4D4G)	Rabbit	MC	Transcription factor regulating interferon (IFN) in response to viral infection	1:1000	Cell Signalling 4947
Anti-cGAS	Rabbit	PC	Active state of cGAS produces the second messenger cyclic GMP–AMP (cGAMP)	1:1000	Abcam (Ab224144)
Anti-NAK/TBK1	Rabbit	MC	Kinase, with a role in innate immunity antiviral response	1:1000	Cell Signaling 3504
Anti-ICAM	Rabbit	MC	Adhesion molecule involved in the transmigration of inflammatory cells through the blood–brain barrier	1:1000	Abcam (Ab53013)
Anti-β-actin	Rabbit	PC	Cytoskeleton	1:3000	Cambridge Bioscience A300-485A
Anti-β-actin	Mouse	MC	Cytoskeleton	1:3000	Merck A5316
			**Secondary antibodies**		
Anti-Mouse IgG Alexa Fluor 647	Goat	PC		1:100	ThermoFisher A-21235

Anti-Rabbit IgG Alexa Fluor 488	Goat	PC	1:100	ThermoFisher A-11034
Anti-Rabbit IRDye® 800CW	Goat	PC	1:15,000	Li-Cor 926-32211
Anti-Mouse IRDye® 800CW	Goat	PC	1:15,000	Li-Cor 926-32210

Abbreviations used: MC; Monoclonal, PC; Polyclonal

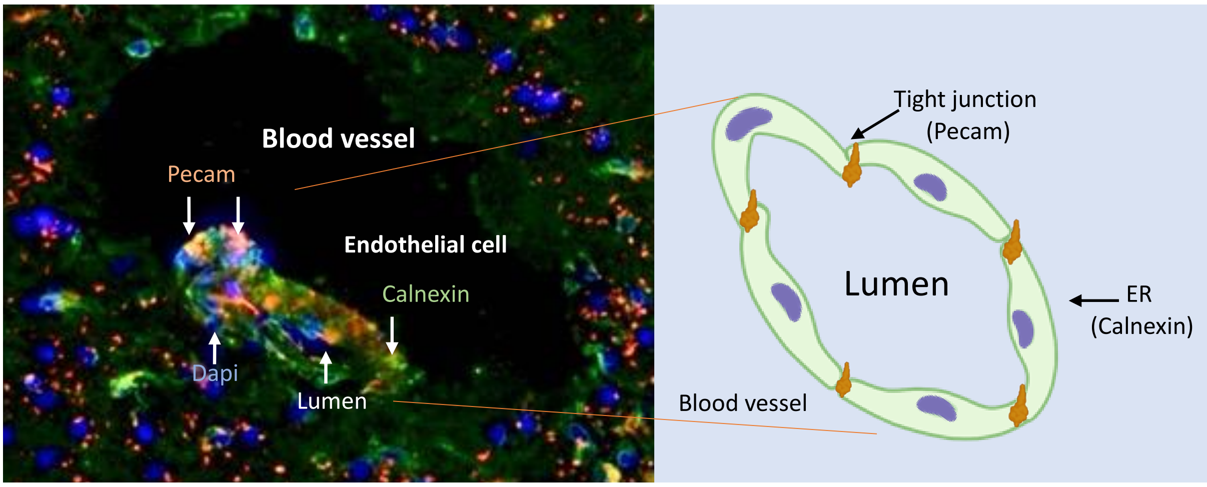

Supplementary Figure S1. Abundant expression of Calnexin in an acute lesional MS blood vessel. Co-staining of PECAM (red) and calnexin (green) throughout ER enriched cytosol in fresh frozen section of white matter from acute region of MS patient brain.

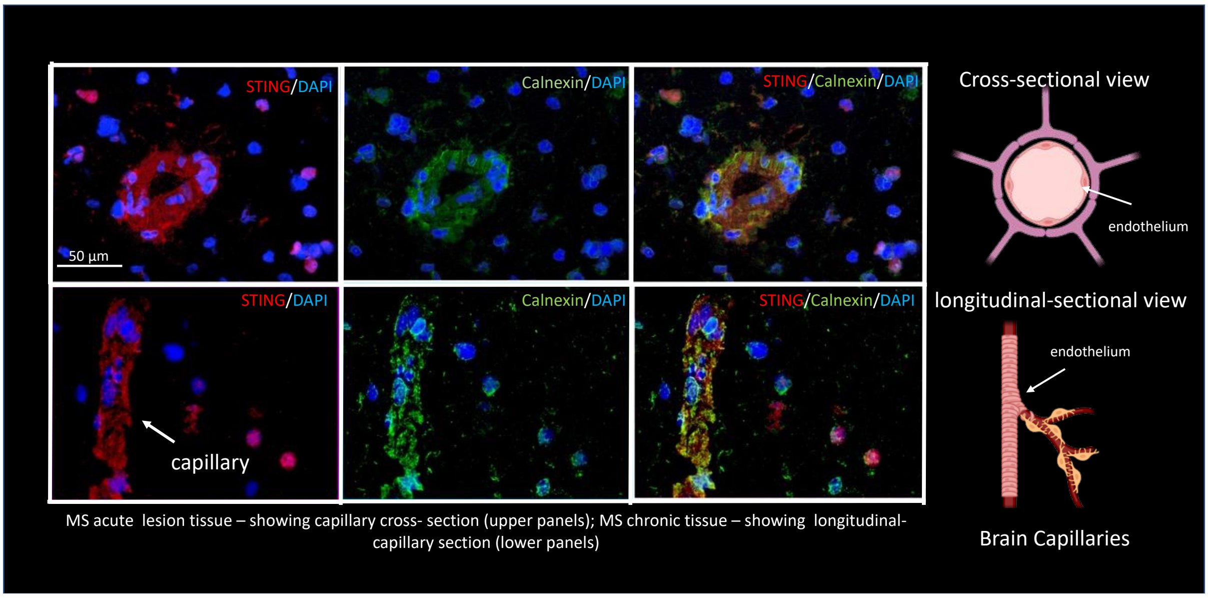

Supplementary Figure S2. Co-staining of calnexin and STING in a cross section and longitudinal section of a blood capillary. Representative images in acute and chronic brain tissue of two MS patients respectively. (Schematic diagrams were drawn using Biorender software).

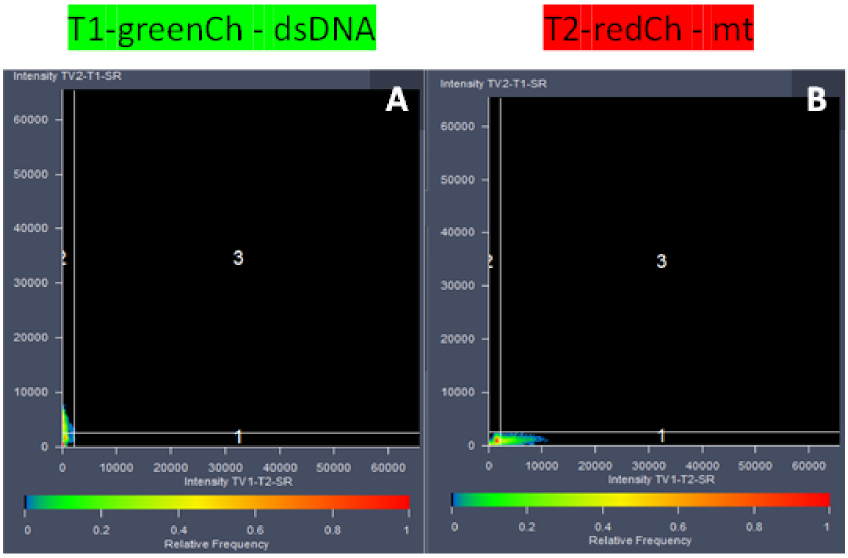

Supplementary Figure S3. Examples of the baseline thresholds set for the colocalization analysis. (**A**) threshold set for the dsDNA channel and (**B**) threshold set for the mt channel. For a full list of the thresholds set see S. Table 3.

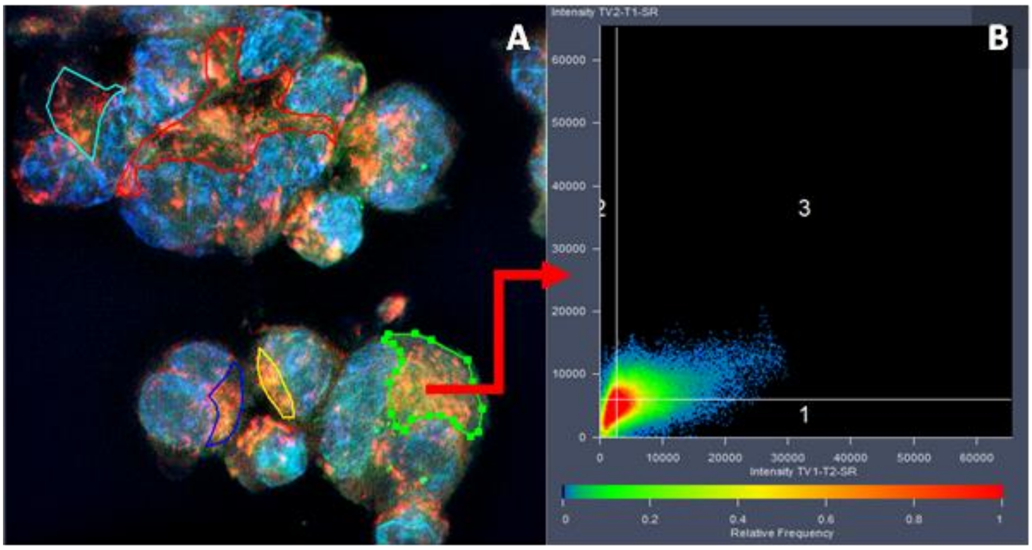

Supplementary Figure S4. Semi-quantification of pixel analysis and cytosolic dsDNA/Mitochondria proximity. (**A**) Illustration of how the cytosolic pixel analysis and the regions of interest (ROI) were selected to generate scatterplots and histogram pixel information. (**B**) A scatter plot example of the proportion of cytosolic dSDNA (from the green ROI), which exhibited a positive correlation with mitochondria. Area1 represents pixel that are red (mt), Area2 represents pixels that are green (dsDNA) and Area3 represents pixels that are collocated (dsDNA-mt).

Supplementary Table S3: The coefficients generated from thresholded quantitative colocalization analysis using IMARIS 9.9. Data presented as means ±SEM (n=7-10).

Descriptor	ctr-ave±sem	100PA-ave±sem	200PA-ave±sem	PA400-ave±sem
threshold A	3443.80±131.04	1699.04±64.17	1321.71±127.48	2358.00±218.50
threshold B	1615.51±139.77	2442.92±447.61	1217.48±230.26	2946.50±79.99
number of colocalized voxels	131931.00±53895.59	502509.20±102012.98	417247.86±107231.75	861135.33±126955.54
% of dataset colocalized	3.14±1.29	11.98±2.43	9.95±2.56	20.53±3.03
% of ROI colocalized	3.14±1.29	11.98±2.43	9.95±2.56	20.53±3.03
% of volume A above threshold colocalized	17.31±3.10	55.16±7.92	70.94±5.51	93.18±1.51
% of volume B above threshold colocalized	39.02±4.83	66.55±5.81	34.37±6.25	48.42±6.21
% of material A above threshold colocalized	16.30±3.30	59.95±8.13	71.62±5.66	94.30±1.20
% of material B above threshold colocalized	38.17±4.85	71.42±5.36	34.11±6.46	52.90±6.17
% of ROI material A colocalized	8.64±2.87	34.07±5.20	42.89±5.94	65.32±4.21
% of ROI material B colocalized	11.66±3.97	35.47±6.21	27.99±5.97	42.52±5.08
Pearson's coefficient in dataset volume	0.43±0.02	0.46±0.01	0.39±0.02	0.66±0.01
Pearson's coefficient in ROI volume	0.43±0.02	0.46±0.01	0.39±0.02	0.66±0.01
Pearson's coefficient in colocalized volume	-0.07±0.02	0.00±0.03	-0.06±0.01	0.17±0.02
original Manders' coefficient A (M1)	0.97±0.01	0.98±0.01	0.97±0.01	1.00±0.00
original Manders' coefficient B (M2)	0.97±0.00	0.97±0.01	0.92±0.01	0.90±0.01
thresholded Manders' coefficient A (M1)	0.13±0.03	0.39±0.06	0.66±0.05	0.89±0.02
thresholded Manders' coefficient B (M2)	0.31±0.05	0.46±0.09	0.30±0.06	0.44±0.05

Regression stats output:

Supplementary Table S4: Regression analysis of the overlapping colocalized pixels (MOC)

```
mod1 <- lm(logcoloc ~ log(conc+1))
summary(mod1)
Call:
lm(formula = logcoloc ~ log(conc + 1))
Residuals:
Min       1Q   Median       3Q      Max
-1.74175 -0.42060 -0.02328  0.45857  1.24248
Coefficients:
Estimate Std. Error Pr(>|t|)
(Intercept)    11.49112    0.28923  39.730  < 2e-16 ***
log(conc + 1)  0.30432    0.06089   4.998 6.03e-05 ***
Signif. codes:  0 '***' 0.001 '**' 0.01 '*' 0.05 '.' 0.1 ' ' 1
Residual standard error: 0.6583 on 21 degrees of freedom
Multiple R-squared:  0.5433,    Adjusted R-squared:  0.5215
F-statistic: 24.98 on 1 and 21 DF,  p-value: 6.025e-05
```

Supplementary Table S5: Regression analysis of the Manders coefficient A (M1) of co-occurrence

```
mod2 <- lm(M1 ~ log(conc+1), data = colocR)
summary(mod3)
Call:
lm(formula = M1 ~ log(conc + 1), data = colocR)
Residuals:
    Min       1Q   Median       3Q      Max
-0.35467 -0.08652  0.06020  0.08880  0.18720
Coefficients:
            Estimate Std. Error t value Pr(>|t|)
(Intercept)    0.08266    0.06984   1.184     0.25
log(conc + 1)  0.11018    0.01470   7.494 2.31e-07 ***
Signif. codes:  0 '***' 0.001 '**' 0.01 '*' 0.05 '.' 0.1 ' ' 1
Residual standard error: 0.1589 on 21 degrees of freedom
Multiple R-squared:  0.7279,    Adjusted R-squared:  0.7149
F-statistic: 56.17 on 1 and 21 DF,  p-value: 2.308e-07
```

Supplementary Table S6: Regression analysis of the Manders coefficient A (M2) of co-occurrence

```
> mod3 <- lm(M2 ~ log(conc+1), data = colocR)
> summary(mod4)
Call:
lm(formula = M2 ~ log(conc + 1), data = colocR)
Residuals:
    Min       1Q   Median       3Q      Max
-0.28348 -0.10465 -0.03507  0.11879  0.35124
Coefficients:
            Estimate Std. Error t value Pr(>|t|)
(Intercept)    0.31247    0.07015   4.454 0.000219 ***
```

log(conc + 1) 0.01412   0.01477   0.956 0.349747

Signif. codes: 0 '***' 0.001 '**' 0.01 '*' 0.05 '.' 0.1 ' ' 1

Residual standard error: 0.1597 on 21 degrees of freedom

Multiple R-squared: 0.04174,   Adjusted R-squared: -0.003892

F-statistic: 0.9147 on 1 and 21 DF, p-value: 0.3497
